# A *Pseudomonas putida* efflux pump acts on short-chain alcohols

**DOI:** 10.1186/s13068-018-1133-9

**Published:** 2018-05-11

**Authors:** Georg Basler, Mitchell Thompson, Danielle Tullman-Ercek, Jay Keasling

**Affiliations:** 1 Department of Chemical and Biomolecular Engineering, University of California, Berkeley, CA USA; 2 Max Planck Institute for Molecular Plant Physiology, Potsdam, Germany; 3 Department of Plant & Microbial Biology, University of California, Berkeley, CA USA; 4 Joint BioEnergy Institute, Emeryville, CA USA; 5 Department of Chemical and Biological Engineering, Northwestern University, Evanston, IL USA; 6 Chemistry of Life Processes Institute, Northwestern University, Evanston, IL USA; 7 Center for Synthetic Biology, Northwestern University, Technological Institute B486, Evanston, USA; 8 Biological Systems and Engineering Division, Lawrence Berkeley National Laboratory, Berkeley, CA USA; 9 Novo Nordisk Foundation Center for Sustainability, Technical University of Denmark, Copenhagen, Denmark

**Keywords:** Higher alcohols, Short-chain alcohols, Next-generation biofuels, Efflux pumps, TtgABC, *Pseudomonas putida*, Tolerance, Toxicity

## Abstract

**Background:**

The microbial production of biofuels is complicated by a tradeoff between yield and toxicity of many fuels. Efflux pumps enable bacteria to tolerate toxic substances by their removal from the cells while bypassing the periplasm. Their use for the microbial production of biofuels can help to improve cell survival, product recovery, and productivity. However, no native efflux pump is known to act on the class of short-chain alcohols, important next-generation biofuels, and it was considered unlikely that such an efflux pump exists.

**Results:**

We report that controlled expression of the RND-type efflux pump TtgABC from *Pseudomonas putida* DOT-T1E strongly improved cell survival in highly toxic levels of the next-generation biofuels *n*-butanol, isobutanol, isoprenol, and isopentanol. GC-FID measurements indicated active efflux of *n*-butanol when the pump is expressed. Conversely, pump expression did not lead to faster growth in media supplemented with low concentrations of *n*-butanol and isopentanol.

**Conclusions:**

TtgABC is the first native efflux pump shown to act on multiple short-chain alcohols. Its controlled expression can be used to improve cell survival and increase production of biofuels as an orthogonal approach to metabolic engineering. Together with the increased interest in *P. putida* for metabolic engineering due to its flexible metabolism, high native tolerance to toxic substances, and various applications of engineering its metabolism, our findings endorse the strain as an excellent biocatalyst for the high-yield production of next-generation biofuels.

**Electronic supplementary material:**

The online version of this article (10.1186/s13068-018-1133-9) contains supplementary material, which is available to authorized users.

## Background

The high tolerance of several gram-negative bacteria to toxic substances compared to other organisms is attributed to a lower outer membrane permeability, periplasmic and cytosolic enzymatic degradation (e.g., β-lactamases), homeoviscous membrane adaptation, and multidrug efflux pumps [1–3]. The latter have the potential not only to improve cell survival in toxic environments, but also to directly increase yield and productivity of production strains by removing the final product from the cells and facilitating extracellular product recovery [4]. Consequently, the discovery of efflux pumps acting on a target product of interest can help to improve microbial production, particularly of toxic substances, such as antibiotics or biofuels [5–7].

Various strains of *Pseudomonas* are known for their ability to metabolize a number of industrial products and solvents as sole carbon source [8, 9], and to tolerate high concentrations of toxic aromatic compounds [10–13]. The strain *Pseudomonas putida* DOT-T1E was isolated from a wastewater plant in Spain [14], and possesses a number of efflux systems. TtgABC is an efflux system of the resistance-nodulation-cell division (RND) type consisting of the inner membrane protein TtgB, the membrane fusion protein TtgA, and the outer membrane channel TtgC [15, 16]. TtgABC is expressed constitutively [15], but is also assumed to be induced by *n*-butanol [17]. It acts on specific aromatic hydrocarbons, such as toluene, *m*-xylene, and 1,2,4-trichlorobenzene, as well as antibiotics [15], in combination with the efflux systems TtgDEF and TtgGHI [18]. While the action of these and other RND-type efflux systems on antibiotics and aromatic compounds is well-studied, their effect on short-chain alcohols, particularly relevant as next-generation biofuels, is thus far not known.

Moreover, a large-scale screen for identification of bacterial efflux pumps acting on biofuels returned no candidates for the short-chain alcohols *n*-butanol and isobutanol [5]. Together with several other attempts to identify efflux pumps for biofuels [19, 20], this led to the assumption that RND-type efflux pumps do not act on short-chain alcohols [4]. A more recent study applied random mutagenesis to the AcrAB-TolC efflux pump of *E. coli*, leading to the identification of mutations which allow export of *n*-butanol, isobutanol, and *n*-heptanol [21]. The pump was introduced into *n*-butanol-producing *E. coli* and shown to increase titers [6]. Unfortunately, this approach for engineering efflux pumps is tedious and limited by the plasticity of the native pump for broadening its substrate specificity with respect to the target compound. Hence, the discovery of efflux pumps acting natively on a desired target product is a promising alternative approach for improving the tolerance of microbial production strains to support metabolic engineering for biofuel production.

We show that controlled expression of the TtgABC efflux system led to a strong increase in survival rate of *P. putida* DOT-T1E exposed to high concentrations of *n*-butanol, isobutanol, isoprenol, and isopentanol. We observed increased extracellular *n*-butanol concentrations when cells expressing the efflux pump were incubated in *n*-butanol containing media, indicating active efflux. This suggests that the TtgABC efflux pump can be used to improve production of a number of short-chain alcohols. On the other hand, we found that growth rates in *n*-butanol and isopentanol were not increased when expressing the pump, hinting at the limitations of growth assays for the identification of efflux pumps with novel functions. To our knowledge, this is the first report of a native RND-type efflux pump shown to export short-chain alcohols.

## Results

Expression of efflux pumps can be toxic and their operation requires energy [22], which implies a tradeoff between the benefit and burden of pump expression in toxic environments [23]. Therefore, the function of an efflux pump critically depends on the level of expression, which must be fine-tuned to maximize its effect while minimizing the burden. To gain quantitative control of expression and fine-tune expression levels to avoid a negative effect on cell survival from overexpression, we placed the TtgABC operon under control of the l-arabinose inducible promoter *P*_BAD_ on a broad host range plasmid (BBR1) and transformed it into *P. putida* DOT-T1E (“Methods”). The plasmid remained stable in *P. putida* for about 2 days without selective antibiotic while expressing TtgABC (Additional file 1: Figure S1). In *E. coli*, induction via the *P*_BAD_ promoter was shown to result in non-homogenous expression levels, whereby different sub-populations exhibit either high or no expression at intermediate inducer concentrations [24]. Since this phenomenon would have complicated observing an effect of the pump on toxic substances at intermediate expression levels, we first tested whether expression via *P*_BAD_ is homogenous in the case of *P. putida* by placing GFP under control of *P*_BAD_ and determining fluorescence after induction with 0, 1, 10, and 100 mM l-arabinose. Using flow cytometry, we found that GFP fluorescence was quantitative and homogenous (Additional file 2: Figure S2), demonstrating that the promoter can be used efficiently with *P. putida* for fine-tuning of expression levels.

**Fig. 1:**
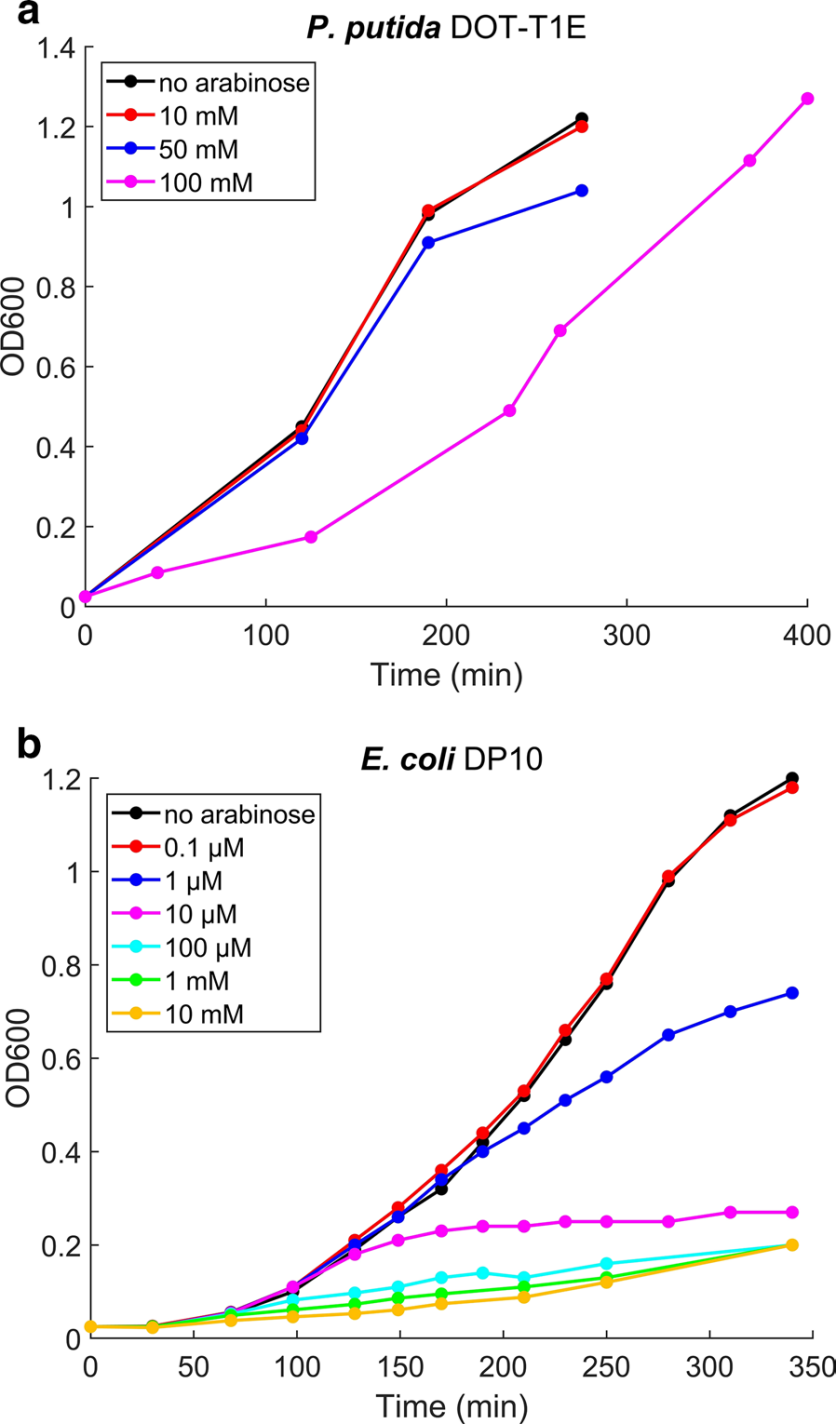
Toxicity of TtgABC expression. The pBbB8k-TtgABC plasmid was introduced into *P. putida* DOT-T1E and *E. coli* DP10, and efflux pump expression was induced using different concentrations of l-arabinose. Growth curves indicate a prolonged lag-phase when inducing expression with 100 mM l-arabinose in *P. putida* (**a**), while growth of *E. coli* is strongly affected starting from inducer concentrations of 1 µM (**b**)

To determine the burden of TtgABC pump expression in *P. putida*, we grew the plasmid-carrying strain with different inducer concentrations. Growth was unaffected when using up to 50 mM of l-arabinose (Fig. 1a). Induction with very high levels of l-arabinose (100 mM) led to a prolonged lag-phase of about 2 h, but the strain then fully recovered growth. For comparison, we expressed TtgABC in an *E. coli* strain engineered to give homogenous expression using *P*_BAD_ [25]. We found that TtgABC expression in this strain inhibits growth starting from l-arabinose concentrations as low as 1 μM (Fig. 1b), indicating that transfer of the pump to other strains may be challenging due to toxicity (see “Discussion”).

We next determined the inducer concentrations, induction times, and growth stage which maximize the effect of TtgABC expression on survival in high concentrations of *n*-butanol (“Methods”). Although previous results indicated that *P. putida* DOT-T1E could grow in the presence of up to 6% *n*-butanol (v/v) after long-term adaptation [12], these results could not be reproduced by us or others [26]. Instead, we observed that the wild-type strain could survive up to 1.9% of *n*-butanol for 2 h, and that its tolerance as measured by growth or survival did not increase even after 2 months of adaptation (“Methods”).

**Fig. 2:**
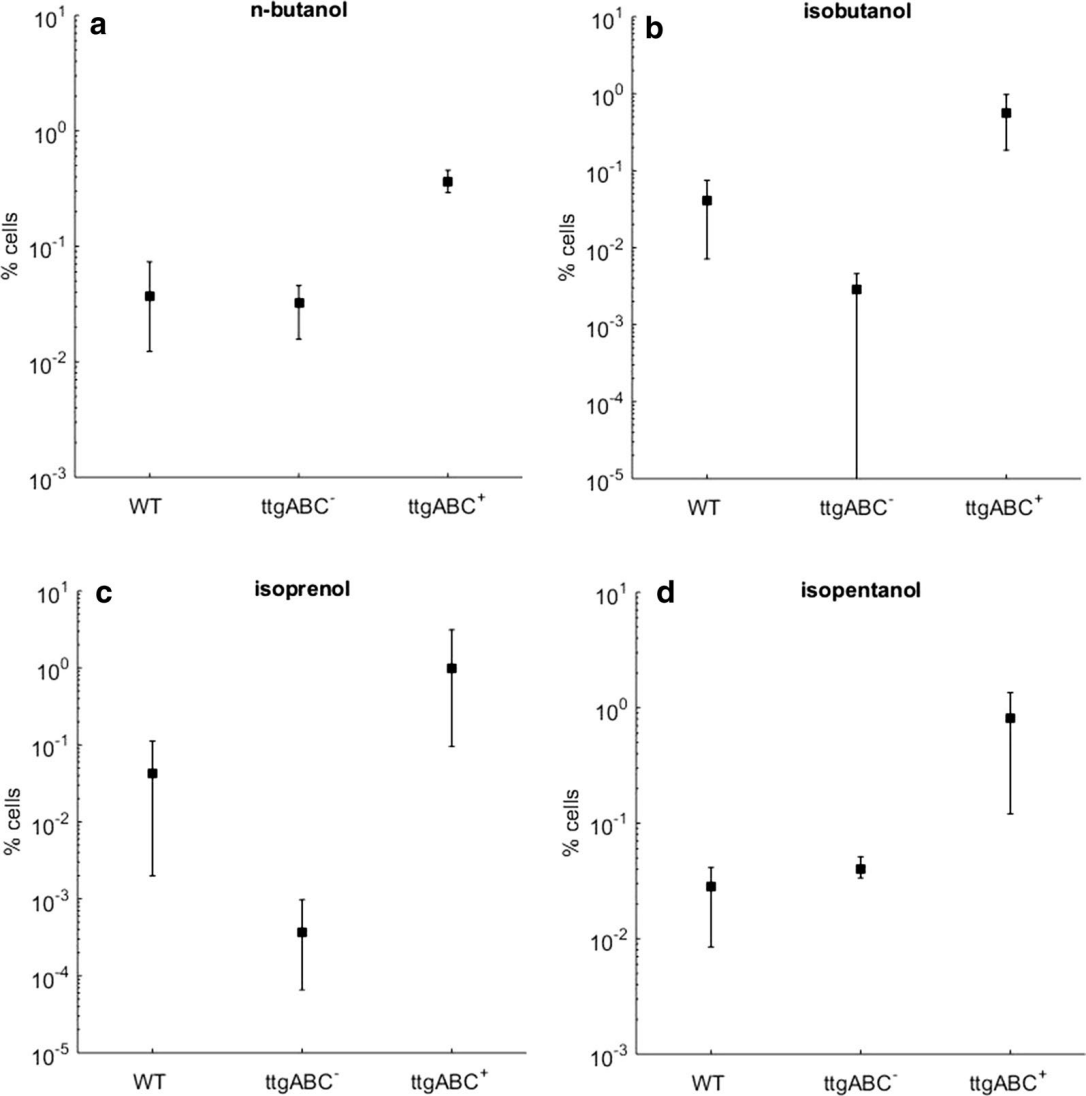
TtgABC expression increases survival of *Pseudomonas putida* DOT-T1E in biofuels. Fraction of *P. putida* DOT-T1E cells surviving 2 h of incubation with **a** 1.9% *n*-butanol **b** 2.2% isobutanol, **c** 1.5% isoprenol, and **d** 0.7% isopentanol. Cells carrying TtgABC on a plasmid with l-arabinose inducible promoter were grown with (ttgABC^+^) or without (ttgABC^−^) inducer. Plasmid-free wild-type cells (WT) were grown with l-arabinose as control. Ratios were obtained by sampling viable cell numbers before and after incubation. The error bars indicate minimum and maximum of 3–6 biological replicates

The TtgABC plasmid-carrying strain was then grown with and without induction, and the plasmid-free wild-type strain was grown with l-arabinose as additional control. Induction of TtgABC expression with 1.5 mM l-arabinose and growth to stationary phase resulted in the largest increase of survival rate, and these conditions were chosen for all subsequent experiments. Note that TtgABC expression did not affect growth up to 50 mM l-arabinose (cf. Fig. 1a). After normalizing the cell densities, cells were incubated with 1.9% *n*-butanol, 2.2% isobutanol, 1.5% isoprenol, or 0.7% isopentanol for 2 h, which represent slightly sub-lethal concentrations. Survival rates were calculated by sampling the number of living cells before and after incubation (Fig. 2, “Methods”). For isobutanol and isoprenol, survival of the wild-type was at least an order of magnitude higher compared to the plasmid-carrying, non-induced variants. This indicates that the presence of the plasmid may decrease survival, which could limit the observable effect in strains expressing the pump. For each of the four alcohols tested, survival of the cells expressing TtgABC was at least tenfold higher compared to the non-induced and wild-type strains.

To assess whether the observed increase in survival was indeed due to efflux of the alcohol, rather than a side effect or general stress response due to expressing the membrane protein, we compared extracellular *n*-butanol concentrations of non-induced and induced cells after incubation for 110 min with 0.2% *n*-butanol via gas chromatography with a flame ionization detector (GC-FID, “Methods”). The rationale for this approach is that strains with higher efflux accumulate less intracellular *n*-butanol, leading to higher measurable extracellular concentrations. The approach was previously used to measure *n*-butanol efflux [21]. We found a significant increase of 5.8% (*t* test, *p* value < 0.05) in extracellular *n*-butanol concentrations of induced cells, which corresponds to about 2.9 × 10^−8^ mol gDW^−1^ s^−1^ more efflux of *n*-butanol from induced cells compared to non-induced cells. We determined the viable cell numbers before and after incubation to ensure that cells were not dying, since this could have led to a release of the alcohol due to membrane disintegration (“Methods”). Hence, our results indicate active efflux of *n*-butanol in cells expressing TtgABC.

The inner membrane pump of TtgABC is TtgB, which is the component responsible for substrate binding. TtgB is homologous to AcrB in the AcrAB-TolC efflux pump of *E. coli* (65% amino acid sequence similarity). Three individual point mutations of AcrB (M355L, I466, S880P) were previously shown to allow export of *n*-butanol, isobutanol, and *n*-heptanol in *E. coli* [21]. Sequence alignment revealed that the same amino acids are present at the corresponding loci of TtgB, with strongly conserved regions surrounding M355 and I466. To test whether increased efflux of *n*-butanol could be achieved in TtgABC, we applied each of the three point mutations, as well as the combination of the three, at the corresponding loci of TtgB (“Methods”). We found that each of the three mutations, as well as their combination, resulted in a decrease of survival rate compared to the wild-type strain (not shown). This may indicate structural differences of the inner membrane pumps, despite their homology, or a generally higher specificity of TtgB to *n*-butanol, compared to AcrB, preventing a further increase of *n*-butanol efflux in TtgABC.

**Fig. 3:**
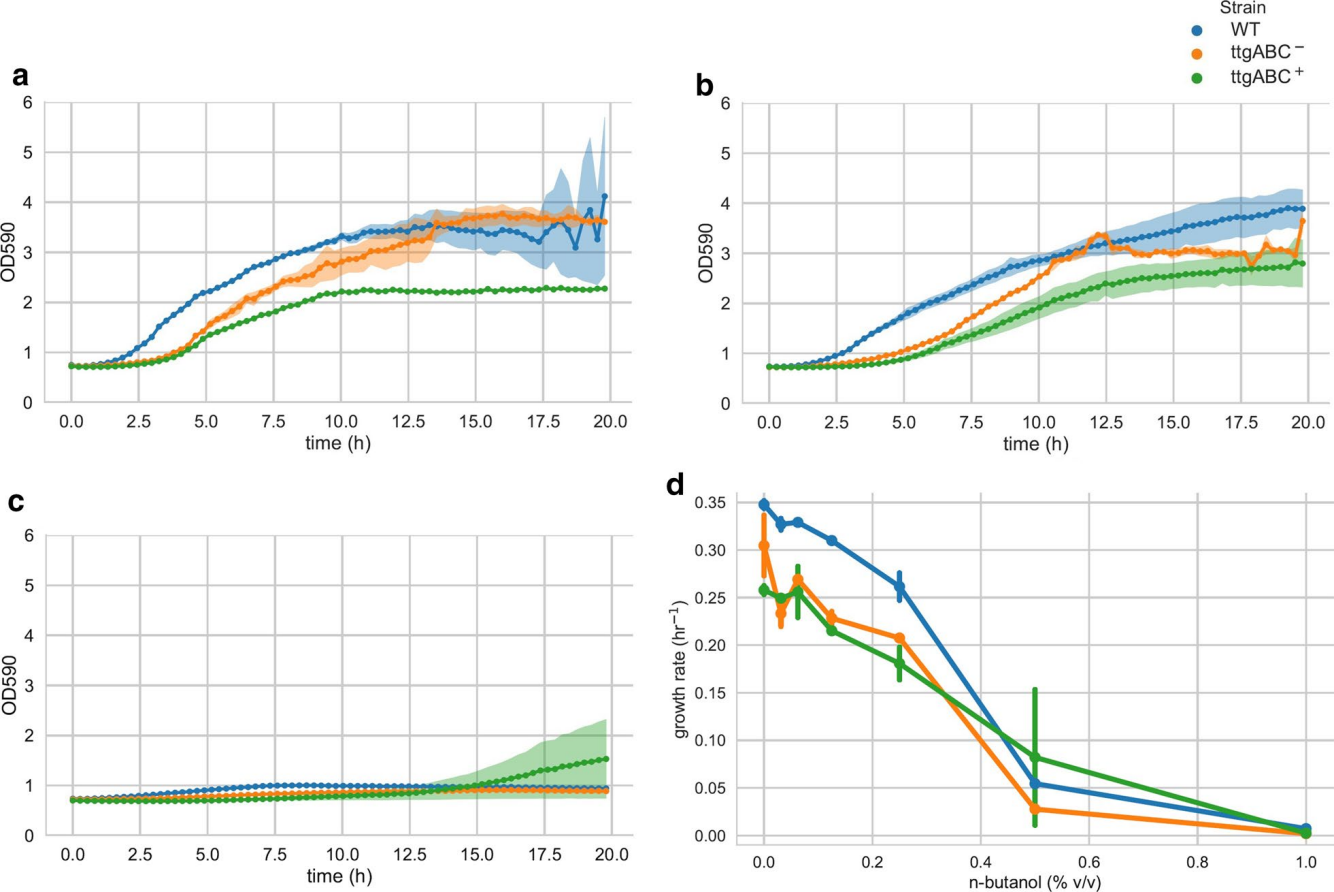
TtgABC expression does not increase growth of *P. putida* DOT-T1E in *n*-butanol. Growth of *P. putida* DOT-T1E without plasmid (WT), without induction (ttgABC^−^), and with induction (ttgABC^+^) in 0% (**a**), 0.25% (**b**), and 0.5% (**c**) *n*-butanol. Growth rates of the wild-type strain are slightly higher, and there is no increase in growth rate due to TtgABC expression (**d**)

**Fig. 4:**
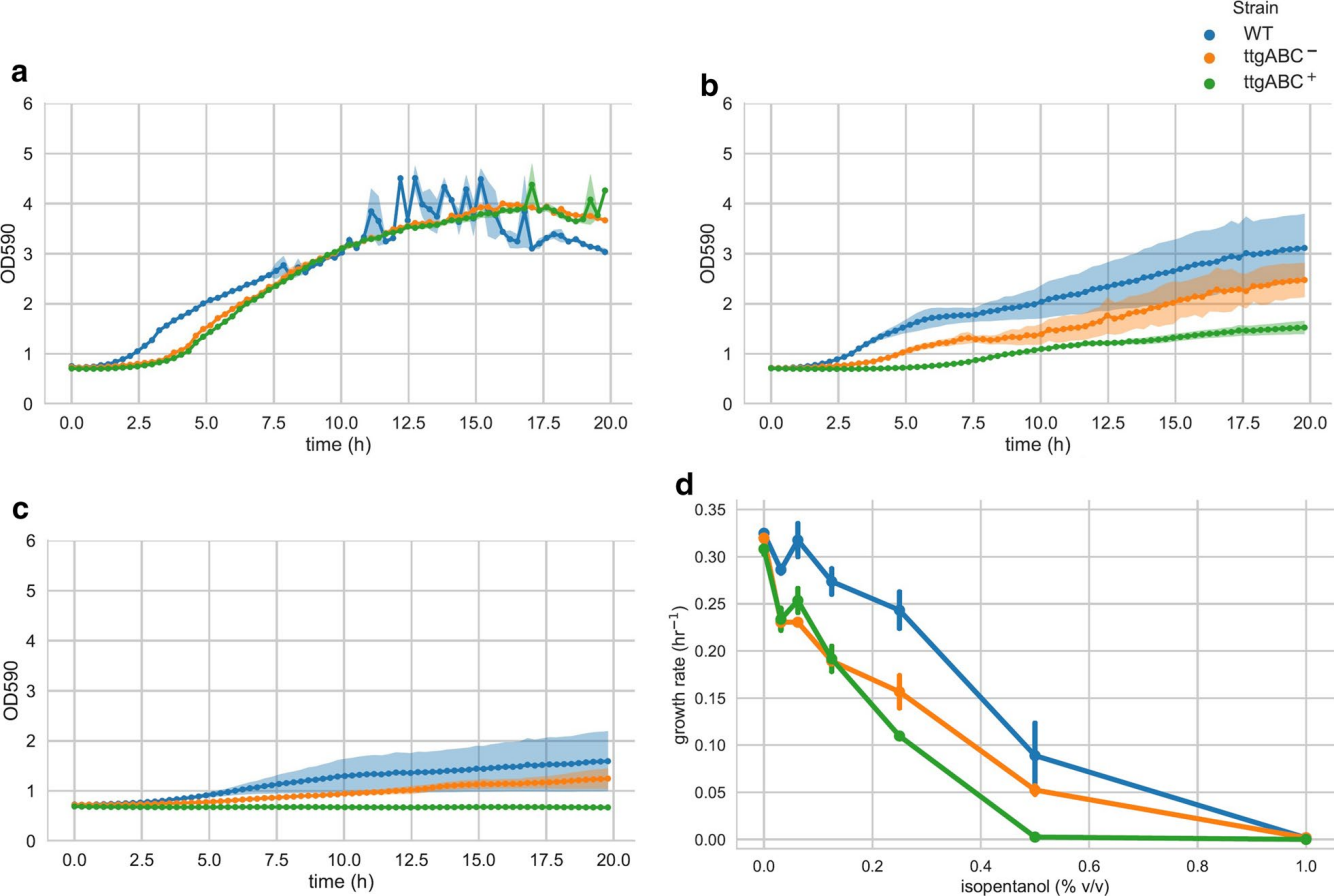
TtgABC expression does not increase growth of *P. putida* DOT-T1E in isopentanol. Growth of *P. putida* DOT-T1E without plasmid (WT), without induction (ttgABC^−^), and with induction (ttgABC^+^) in 0% (**a**), 0.25% (**b**), and 0.5% (**c**) isopentanol. Growth rates of the wild-type strain are slightly higher, and there is no increase in growth rate due to TtgABC expression (**d**)

To assess whether the effect of efflux pump expression on cell survival implies faster growth in biofuels, we grew the induced and non-induced plasmid-carrying strains, as well as the plasmid-free wild-type strain, in a plate reader with a range of *n*-butanol and isopentanol concentrations (Figs. 3, 4). Growth rates were largely unaffected up to concentrations of 0.125% (v/v), and the wild-type strain grew slightly faster (0.26–0.35 h^−1^) compared to non-induced (0.19–0.34 h^−1^) and induced (0.18–0.31 h^−1^) plasmid-carrying strains in both alcohols. The effect was even more pronounced at 0.25% (v/v), where growth of all strains was slightly affected, and the wild-type strain grew faster (0.23–0.28 h^−1^) compared to the non-induced (0.14–0.21 h^−1^) and induced (0.11–0.2 h^−1^) plasmid-carrying strains. Moreover, there was no difference in growth rates between non-induced and induced strains for any of the tested concentrations. To account for the possibility of oxygen limitation in plate readers due to the small volumes used, we also tested growth in larger volumes (Additional file 3: Figure S3, “Methods”). Without alcohol, growth rates were higher compared to the plate reader and similar for all strains (0.53 h^−1^). With 0.5% *n*-butanol, growth rates of the wild-type and non-induced strains were slightly higher (0.29 h^−1^) compared to the induced strain (0.26 h^−1^). Hence, the observed effect of TtgABC on survival in highly toxic conditions does not translate into increased growth in less toxic biofuel concentrations. Accordingly, growth assays could not have identified the observed effect of the TtgABC efflux pump on short-chain alcohols.

## Methods

### Plasmid construction and survival assays

We amplified *ttgABC* (5762 bp) from the genome of *P. putida* DOT-T1E and cloned it on a broad host range plasmid (BBR1) between the l-arabinose inducible *P*_BAD_ promoter and double terminator using Gibson Assembly. The final construct, termed pBbB8k-TtgABC after the Bgl-Brick standard [27] has a length of 9529 bp and further contains *araC* and Kanamycin resistance marker. For measuring expression levels, we created another plasmid containing GFP between the *P*_BAD_ promoter and double terminator. The plasmids were transformed into *P. putida* DOT-T1E and *E. coli* DP10 [25] using electroporation (Bio-Rad MicroPulser).

For the survival assays, three to six colonies were inoculated in LB medium with 1.5 mM (induced) and without (non-induced) l-arabinose and incubated shaking at 200 rpm and 30 °C overnight. After reaching stationary phase, all samples were diluted to an equal optical density of 0.1 at 600 nm, and 2 mL of diluted culture (approximately 1.6 × 10^8^ cells) was incubated shaking at 200 rpm and 30 °C in an airtight 15-mL plastic tube with the corresponding alcohol for 2 h. Diluted cell cultures were plated on selective medium (plasmid-carrying variants) and LB (plasmid-free WT) before and after incubation, and colonies were counted to determine the percent of surviving cells.

### Long-term adaptation of *P. putida* to *n*-butanol

*Pseudomonas putida* DOT-T1E was grown for 2 months in 20 mL LB medium with 33 mM l-arabinose, 0.5% *n*-butanol, and 50 µg/mL rifampycin. The medium was renewed daily by sub-culturing of 100 µL into fresh medium. After 1 month, the strain was split into two cultures. One culture was continued with 0.5% *n*-butanol in the medium, while the other was exposed to increasing concentrations of up to 1% *n*-butanol, with renewal of medium after reaching a visible density (approximately every 3 days). After 2 months, the strains were exposed to a range of *n*-butanol concentrations to test for increased tolerance. No difference was observed in survival or growth between the strains undergoing the long-term adaptation and the original wild-type strains.

### Quantification of extracellular *n*-butanol using GC-FID

Three biological replicates were grown overnight with and without 1.5 mM l-arabinose as described in “Plasmid construction and survival assays”. Cell numbers were equalized by determining optical density and transferring an adjusted volume of ~ 50 mL at OD600 of 1.38 into centrifuge falcons. Assuming 1 OD600 unit corresponds to 8 × 10^8^ cells mL^−1^, this density corresponds to about 5.5 × 10^10^ cells. Samples were split in half and cultures were centrifuged three times at 4000×*g* and washed with PBS at pH = 6.5. The last pellet was re-suspended in PBS with 0.2% *n*-butanol to reach a final weight of 1 g, roughly half of which were cells. Cells were incubated at room temperature for 110 min, and then centrifuged at 4000×*g*. 250 µL of supernatant was added to 250 µL of ethyl acetate with isoprenol as internal standard, vigorously vortexed for 15 min, and centrifuged at 22×*g* for 1 min. 100 µL of the organic phase was then removed for further analysis.

Alcohols were quantified using a Tr-Wax column (0.25 mm by 30 m, 0.25-μm film thickness; Thermo Electron) on a Focus GC apparatus with a TriPlus autosampler (Thermo Electron). Hydrogen was used as a carrier, and was set at a constant pressure of 300 kPa, with the inlet temperature set to 200 °C. The oven program was 40 °C for 1.5 min and then it was increased from 40 to 110 °C at 15 °C min^−1^. Samples were normalized using isoprenol as internal standard and quantified using authentic standards. The measurements of the three replicates of induced and non-induced strains are shown in Additional file 4.

To test whether cells were dying during incubation, diluted cell cultures were plated before and after incubation with 0.2 and 1% *n*-butanol, and the resulting colonies counted. Although we observed a slight decrease in cell numbers, this is likely due to a loss of sample when extracting the supernatant, because cell counts were similar at 0.2 and 1% *n*-butanol (Additional file 4). Moreover, there was no significant difference in cell counts between induced and non-induced cells after incubation (*t* test, *p* value 0.26), indicating that pump expression does not lead to cell death under these conditions.

### Transfer of point mutations from AcrB to TtgB

Three mutations of AcrB were previously shown to individually increase efflux of *n*-butanol, isobutanol, and *n*-heptanol [21]. We applied the three point mutations M355L, I466F, and S878P (corresponding to S880P in the aligned AcrB) individually, and the combination of the three, to TtgB by PCR amplification with mismatch primers and Gibson Assembly of the resulting amplicons. The created constructs pBbB8k-TtgABC[M355L], pBbB8k-TtgABC[I466F], pBbB8k-TtgABC[S878P], and pBbB8k-TtgABC[M355L,I466F,S878P] were sequenced and transformed into *P. putida* DOT-T1E via electroporation. The strains were then subjected to the survival assay for *n*-butanol described in “Plasmid construction and survival assays”.

### Growth curves

Growth rates were estimated via a microplate reader kinetic assay. Overnight cultures of *P. putida* grown in LB medium at 30 °C were diluted 1:100 into fresh LB media with either 1.5 mM (induced) or 0 mM (non-induced) l-arabinose and various concentrations of twofold diluted *n*-butanol or isopentanol in 96-well plates (Falcon, 353072). Plates were sealed with a gas-permeable microplate adhesive film (VWR, USA), and then optical density and fluorescence were monitored for 22 h in an Infinite F200 Pro (Tecan Life Sciences, San Jose, CA) plate reader at 30 °C. Optical density was measured at 590 nm every 15 min. In between reads, the plate was shaken at a linear amplitude of 6 mm. All data were analyzed using custom Python scripts. Growth rates were calculated via a 10-timepoint sliding window on optical densities normalized to a 1-cm path length, where the maximal slope with an *r*^2^ > 0.95 was reported as the maximal growth rate. Growth curves were also done in 15 mL conical tubes containing 3 mL medium (Additional file 3: Figure S3), incubated shaking at 200 rpm at 30 °C. Optical densities for this experiment were measured at 600 nm.

## Discussion

Short-chain alcohols are of great interest as biofuels because of their high energy–density, superior transport capabilities, and possibility of directly replacing common engine fuels as drop-ins. Several engineering strategies demonstrated the successful production of short-chain alcohols, including *n*-butanol, isobutanol, isopentanol, and isoprenol using *E. coli* [28–31]. However, their production is strongly affected by the toxicity of the product, leading to suboptimal yields. Several examples demonstrate that the use of efflux pumps can confer increased product yields [4–7]. For example, expression of the ATP-binding cassette transporter MdlB of *E. coli* increased tolerance to and production of isopentenol, although no effect on other short-chain alcohols was observed [32]. This suggests that MdlB acts on isopentenol, even though efflux was not shown directly.

It was considered unlikely that efflux pumps of the type RND act on short-chain alcohols, because all pumps tested so far did not have an effect on these biofuels [4]. For example, knockout of AcrAB in *E. coli* did not affect cell survival in high concentrations of ethanol or 1-propanol compared to the wild-type strain, regardless of whether or not the pump was induced with the mar phenotype [19]. The authors concluded that the AcrAB-TolC efflux pump does not act on short-chain alcohols. Similarly, although mutations in AcrA increased tolerance of *E. coli* to isobutanol, measured by optical density of the cultures after treatment with the alcohol for 24 h, its knockout reduced tolerance [20]. This suggests that AcrA does not have a (positive) effect on isobutanol tolerance with respect to growth.

Moreover, in a large-scale screening approach [5], 43 efflux pumps, including TtgABC and AcrAB-TolC, were tested for their effect on seven relevant biofuels. All pumps were cloned into *E. coli*, and the resulting library of strains was challenged with the biofuels resulting in competition for growth. Even though several efflux pumps acting on other biofuels were identified by the study, no pump was found to act on *n*-butanol or isopentanol. Specifically, TtgABC was found not to have an effect on these compounds. However, the study differs from the approach presented here in two important aspects: first, *E. coli* was used as a host to express the heterologous pumps, and second, growth, rather than survival, was used to assess biofuel toxicity. We found that expression of TtgABC in *E. coli* inhibits growth (Fig. 1b), indicating that toxicity of the heterologous pump could have masked its effect on biofuel tolerance. Moreover, we also did not observe any effect of the pump on growth of *P. putida* in medium supplemented with low concentrations of biofuels (cf. Fig. 3). Hence, it is likely that the function of the pump is to improve survival under highly toxic conditions, rather than enabling faster growth. Consequently, a competitive growth assay could not have identified the effect of the TtgABC pump on biofuels.

We expect that the production and recovery of the short-chain alcohols *n*-butanol, isobutanol, isopentanol and isoprenol can be directly improved by expressing TtgABC in the production strain. We only tested these four short-chain alcohols here, and found that TtgABC acts on each of them. It is therefore likely that the pump also acts on other short-chain alcohols, many of which are promising biofuel candidates: for example, the structurally similar 1-propanol, isopropanol, 1-pentanol, prenol, and 2-methyl-1-butanol have been successfully produced with engineered bacteria [28, 33–35]. To our knowledge, no efflux pump is known yet to act on these compounds, and TtgABC would be a promising candidate to test for decreasing toxicity and improving their production.

The observed toxicity of expressing the pump in *E. coli* (cf. Fig. 1b) indicates that directly transferring TtgABC to other strains may be challenging. Recently identified mutations in *E. coli* leading to a reduced burden of expressing membrane proteins may help to reduce the toxicity of the efflux pump [36], as it may chaperone overexpression [37]. Strategies for regulating membrane protein production via genetic circuits are also promising [6]. Moreover, *P. putida* may itself serve as production host, and several studies have already demonstrated that the strain can be engineered as a robust biocatalyst. Plenty of genetic tools are available for modifying the genetic potential of the strain [38–40]. It has been metabolically engineered for the production of a large variety of compounds, including polyketides, non-ribosomal peptides, rhamnolipids, and aromatic and non-aromatic compounds (reviewed in [41]). The strain has also great potential for the direct production of biofuels from lignocellulosic biomass, as it has been successfully engineered for production of aromatic compounds from lignin components [42, 43]. The strain was also successfully engineered to grow under anaerobic conditions [44–46], and it was used in two-phase liquid extraction systems [47, 48], facilitating the production and extraction of toxic apolar compounds in bioreactors.

More specifically, *P. putida* was engineered for production of *n*-butanol by introducing the biosynthetic pathway from *C. acetobutylicum* [49]. Since *P. putida* degrades *n*-butanol [13], it will be important to knock out the corresponding genes to further increase yields [50]. Hence, the next step will be to express TtgABC in a production strain deficient of *n*-butanol consumption and determine the increase in yield. Since we observed a potential burden of the introduced plasmid on survival and growth (cf. Figs. 2b, c), an alternative approach would be to modify the regulatory region of TtgABC on the genome to obtain control over efflux pump expression without the need of a plasmid, e.g., using classical transposon-based techniques [40]. Although a recent study indicates that genome editing using lambda Red based recombineering of *P. putida* KT2440 is possible, our attempts with the strain DOT-T1E were not successful. An alternative approach would be to identify plasmids which do not affect the strain when exposed to toxic substances, such as plasmids shown to represent a minimal burden for growth in several strains of *Pseudomonas* [51]. With the help of such approaches, the effect of TtgABC expression on tolerance to short-chain alcohols can be further optimized with the aim of maximizing yields in biofuel production strains.

## Conclusions

We showed that the TtgABC efflux pump in *P. putida* DOT-T1E improved tolerance to four short-chain alcohols and increases efflux of *n*-butanol. It is likely that the efflux pump acts on other yet undetermined compounds of industrial relevance, particularly short-chain alcohols. Controlled expression of the efflux pump and identification of the conditions which maximize its effect on tolerance open the possibility of directly testing the effect of the pump on other toxic substances, further engineering its substrate specificity, and its use in metabolic engineering applications.

Moreover, we demonstrated that the specific conditions and levels of pump expression, as well as the type of assay used to determine toxicity, can have a great influence on the observed effect of the efflux pump. Hence, our results indicate that the in-depth study of an individual efflux pump, in addition to large-scale screening approaches, may lead to the identification of efflux pumps with novel functions. The techniques employed here are easy to implement and can be readily applied to other strains and efflux pumps. Consequently, it is possible that novel efflux pump functions for use in microbial production of biofuels or other toxic substances will be discovered using the presented approach.

This is the first report of a native RND-type efflux pump acting on short-chain alcohols, an important class of next-generation biofuels. Together with the availability of genetic tools, catabolic diversity, versatile metabolism and high intrinsic tolerance of the strain to various toxic substances [52], we believe that *P. putida* will become an important biocatalyst for biofuels. To this end, the TtgABC efflux pump will provide a valuable tool for reducing toxicity and increasing yields.

## Additional files

**Additional file 1: Figure S1.** Plasmid stability of pBbB8k-TtgABC in *Pseudomonas putida* DOT-T1E. The plasmid-carrying strain was grown for 5 consecutive days with 2 mM L-arabinose for inducing expression of TtgABC and without kanamycin. The medium was renewed once per day. Plasmid stability was determined by plating on kanamycin plates and comparison of viable cell numbers.

**Additional file 2: Figure S2.** Cell-level GFP expression using the L-arabinose inducible P_BAD_ promoter in *Pseudomonas putida* DOT-T1E. Cells carrying the pBbB8k-GFP plasmid were induced overnight without (brown), with 1 mM (red), 10 mM (purple), and 100 mM (green) L-arabinose. Cell-level fluorescence of GFP was measured using flow cytometry. The distributions indicate a homologous and quantitative increase of expression at the cell level.

**Additional file 3: Figure S3.** TtgABC expression does not increase growth of *P. putida* DOT-T1E in n-butanol. Growth of *P. putida* DOT-T1E without plasmid (WT), without induction (*ttgABC*^*-*^), and with induction (*ttgABC*^*+*^) in 0% **(a)** and 0.5% **(b)** n-butanol in 15 mL conical tubes containing 3 mL medium. Similar to the results obtained from the plate reader (cf. Fig. 3), growth of the wild-type strain is slightly faster with and without n-butanol, and TtgABC expression does not increase growth in n-butanol.

**Additional file 4.** Quantification of extracellular n-butanol using GC-FID. The spreadsheet contains the estimated fraction of viable cells after incubation in PBS containing 0.2 and 1% n-butanol, and the measurement values of quantified extracellular n-butanol in induced (*ttgABC*^*+*^) and non-induced strains (*ttgABC*^*-*^). The original readings for n-butanol and the internal standard isoprenol are shown, as well as the n-butanol measurements normalized by the standard, and the derived concentrations as volume per volume and milligrams per milliliter. The inlay shows the n-butanol standard curve, indicating that concentrations are in a linear measurement range.
