# Supplementary figures and images for: A Pseudomonas putida efflux pump acts on short-chain alcohols
Source: Biotechnol Biofuels. 2018 May 11;11:136. doi: 10.1186/s13068-018-1133-9 (PMC5946390; doi:10.1186/s13068-018-1133-9)

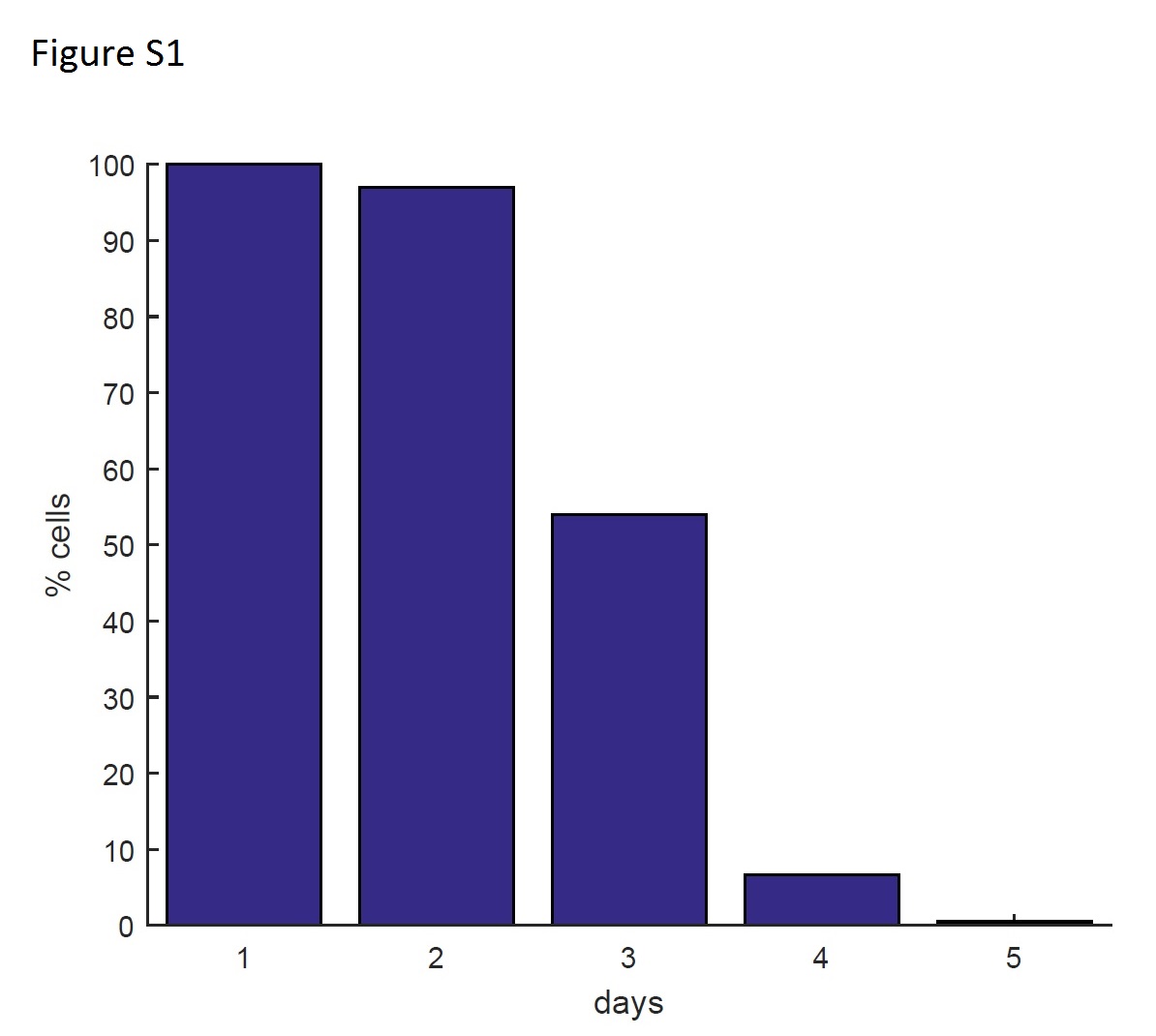

Supplement: Supplementary file 1 — Additional file 1: Figure S1. Plasmid stability of pBbB8k-TtgABC in Pseudomonas putida DOT-T1E. The plasmid-carrying strain was grown for 5 consecutive days with 2 mM L-arabinose for inducing expression of TtgABC and without kanamycin. The medium was renewed once per day. Plasmid stability was determined by plating on kanamycin plates and comparison of viable cell numbers. [file 13068_2018_1133_MOESM1_ESM.jpg]

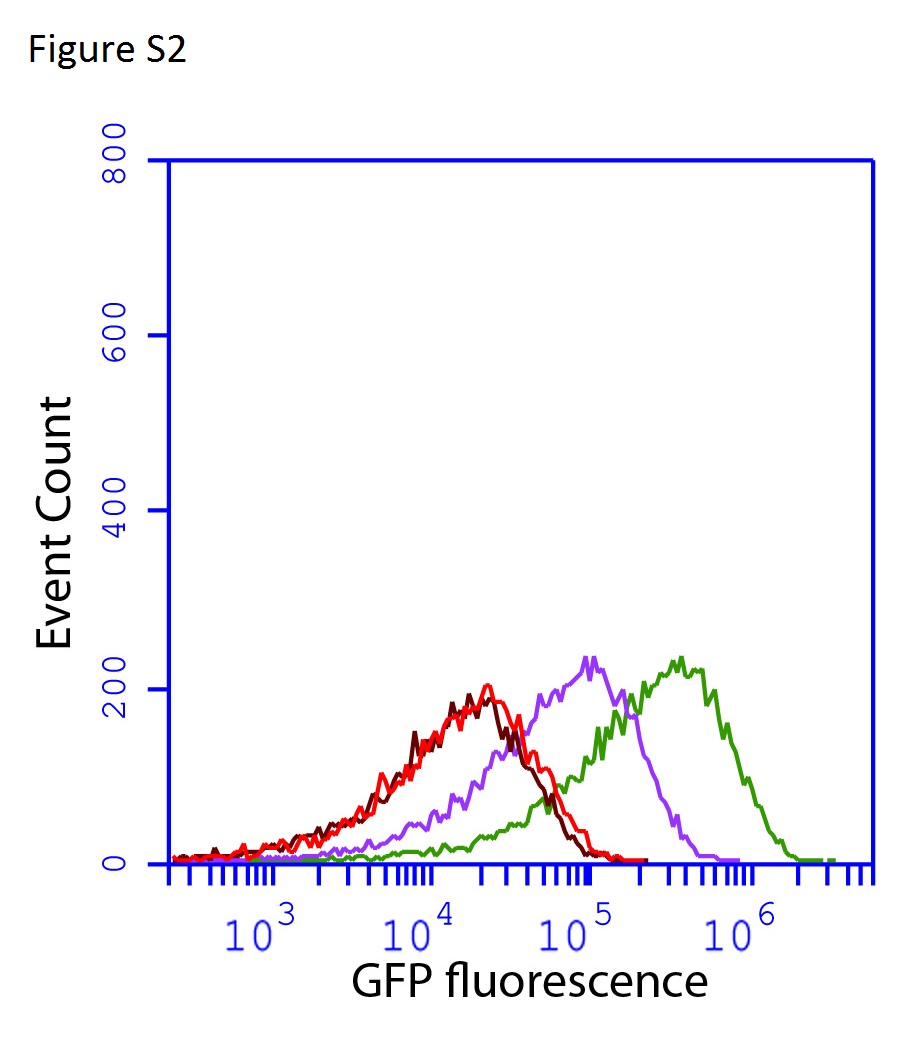

Supplement: Supplementary file 2 — Additional file 2: Figure S2. Cell-level GFP expression using the L-arabinose inducible PBAD promoter in Pseudomonas putida DOT-T1E. Cells carrying the pBbB8k-GFP plasmid were induced overnight without (brown), with 1 mM (red), 10 mM (purple), and 100 mM (green) L-arabinose. Cell-level fluorescence of GFP was measured using flow cytometry. The distributions indicate a homologous and quantitative increase of expression at the cell level. [file 13068_2018_1133_MOESM2_ESM.jpg]

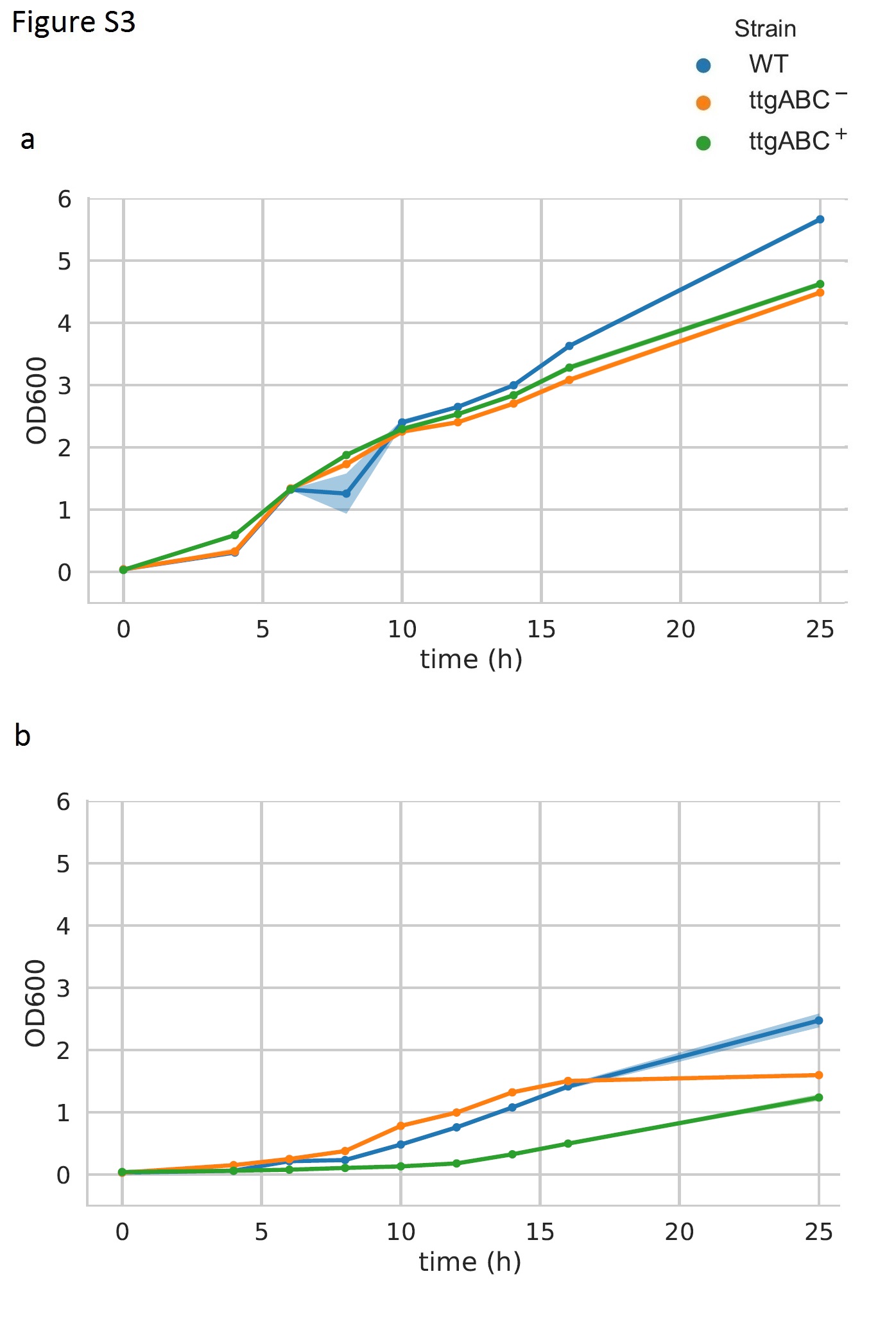

Supplement: Supplementary file 3 — Additional file 3: Figure S3. TtgABC expression does not increase growth of P. putida DOT-T1E in n-butanol. Growth of P. putida DOT-T1E without plasmid (WT), without induction (ttgABC-), and with induction (ttgABC+) in 0% (a) and 0.5% (b) n-butanol in 15 mL conical tubes containing 3 mL medium. Similar to the results obtained from the plate reader (cf. Fig. 3), growth of the wild-type strain is slightly faster with and without n-butanol, and TtgABC expression does not increase growth in n-butanol. [file 13068_2018_1133_MOESM3_ESM.jpg]
